# The utility of innominate dry bone weight in age estimation: A new pelvic aging method

**DOI:** 10.1371/journal.pone.0350291

**Published:** 2026-05-29

**Authors:** Andrea M. Zurek-Ost, Mark Sorensen

**Affiliations:** 1 Research Laboratories of Archaeology, University of North Carolina at Chapel Hill, Chapel Hill, North Carolina, United States of America; 2 Anthropology Department, University of North Carolina at Chapel Hill, Chapel Hill, North Carolina, United States of America; University of Szeged Institute of Biology: Szegedi Tudomanyegyetem Biologia Intezet, HUNGARY

## Abstract

This research investigated the relationships between bone weight, chronological age, and biological sex. This study examined 383 innominates from individuals aged 16–93 years to investigate how bone weight (here a proxy for bone mineral density) was patterned throughout the lifespan. Standard osteology laboratory equipment as well as a scale were used to measure innominate height and dry bone weight. Dry bone weight is an imperfect proxy for bone mineral density; however, the phenomenon of decreases in bone weight with age was observed and quantified in this dry bone sample. While both males and females are affected by a slow reduction in bone mineral density over the course of their lifespans, this effect is exaggerated in females and was reflected in the study results. To quantify these changes, the authors created predictive Bayesian linear models to estimate age at death from dry bone weight, height, and biological sex. The best performing model R^2^ was 0.33, with an average inaccuracy of 9.37 years. Estimated age was within ±10 years of known age for 57.3% of the training sample, and 87.2% of estimated ages at death were within ±20 years of known age. When these models were tested on a smaller modern subsample, the results were both less accurate and less precise, demonstrating a need for validation on expanded modern samples. Overall, these results demonstrate that significant decreases in dry bone weight are associated with increased chronological age in the training sample, particularly in females. This study demonstrates that dry bone weights may be used to support estimates of chronological age; however, they should be applied cautiously, further tested, and only used in concert with well-validated age-at-death estimation methods.

## Introduction

An understanding of age-related changes throughout the lifespan is important for both studies of senescence in living individuals and for the process of postmortem identification of unidentified decedents. Age has long been considered one of the most complex aspects of the biological profile, and while there is a great body of clinical literature regarding the aging process, changes seen in living individuals are not always clearly defined in the skeletal record [1]. Furthermore, skeletal evidence of aging can be difficult to observe consistently and expediently, and the difficulties in identification of highly age-dependent morphological features has long been the subject of osteological inquiry [1, 2].

Although age-at-death estimation methods from skeletal indicators such as the auricular surface and cranial sutures do not always treat males and females as aging differently from one another, clinical literature indicates that skeletal aging patterns in older females differ substantially from than those in older males [3, 4]. Age changes in the osteological record can be difficult to quantify and score consistently, particularly between males and females, because the timing of changes in specific morphological features is not well understood [5]. There are, however, changes to bone which have been related to age, show differences between males and females, and which do not involve the visual inspection and scoring of specific morphological traits. One of these is changes in bone mineral density over time. Bone mineral density change with age is influenced by sex specific patterning [6]. Studies indicate peak bone mass and size (up to 90%) occurs in women around 18 years of age, and in men around 20 years of age [7].

Following this period, bone mass is influenced by a number of factors, including calcium intake and absorption, parity, endocrine status, nutrition, hormones such as estrogen, and body mass [8, 9]. Bone mineral density also decreases with age, in part because of a significant reduction in bone formation as age increases [10]. While the literature reports that both males and females are affected by a slow reduction in bone mineral density over the course of their life spans, this effect is exaggerated in women [11,12]. The NIH Osteoporosis and Related Bone Diseases National Resource Center reports that women tend to experience a change in total bone mass between age 30 and the time of menopause which is relatively minimal; however, the onset of menopause precipitates low levels of estrogen, accelerating bone loss [7,10,11]. An estimated 43.1 million Americans and 200 million people worldwide, both males and females, have low bone mineral density [9].

A decrease in the integrity of the composition, structure, and function of bone with age can lead to osteoporosis. Osteoporosis is characterized by a decrease in bone mineral density and bone mass, as well as concomitant bone structural changes [13]. Osteoporosis is influenced by the reduction of osteoblastic activity and the increase in osteoclastic activity, a shift which occurs with increased age [10]. Osteoporosis affects both males and females but is four times more common in women age 50 or older than in men [14]. This condition is also reportedly more common in white Americans than in black Americans [9].

While these changes have been explored in biological anthropological and clinical literature, they have also been explored in the osteological literature concerning dry bone. For example, Hale and Ross [15] utilized dual-energy X-ray absorptiometry (DXA) to scan dry bone in forensic contexts. As DXA scans assess bone mineral density, they can be useful tools in contexts that may involve starvation, neglect, identification of metabolic bone diseases, and taphonomic studies [15]. DXA scans have also been used to track fracture risk across multiple demographic groups [16].

Less common are the studies in osteological literature which have investigated the potential correlation between age and dry bone weight. The authors were able to identify one method: the recently released age-at-death estimation method Transition Analysis 3 (TA3) that includes dry bone weights for several skeletal elements in the method scoring manual. The definition of this trait, however, is subjective and based on the bone appearing either “normal” or “light.” Normal is defined as “The bone weight falls within what one might expect for an individual of that size. Bones that are borderline, or only slightly lighter than normal, should be scored as normal. This observation is subjective, so err on the side of classifying the bone as normal.” Light is defined as: “The bone must be noticeably lighter than expected, but otherwise normal in appearance. In light innominates, the cortical bone is often extremely fragile and paper-thin.” In the first iteration of the trait scoring manual, the authors noted that “a rough approximation of bone “density” should also be obtained metrically whenever the bone is complete based on: maximum height measured using a standard osteometric board and rounded to the nearest whole millimeter [and] weight measured using a digital scale and recorded to the nearest whole gram.” There were no guidelines associated with this definition to provide guidance to practitioners regarding how to approximate bone density from these traits.

This mention of metrics has been eliminated in the most recent iteration of the TA3 scoring manual [17].

The relationship between innominate size and overall body size has also been noted elsewhere. For example, Auerbach and Ruff [18] investigated the association between biauricular breadth (which necessitates the reassociation of both the right and left innominate) and body mass, when body mass was calculated using both sex-specific and combined-sex formulae. Any studies attempting to estimate age should, given this information, also attempt to account for body size.

Although the incorporation of dry bone weight into analyses of age at death is not a common approach and lacks clear, objective standards when it is present, work of this nature has potential as a cross-disciplinary bridge between age-related changes observed in a clinical setting and those noted in the osteological record.

This research had three primary aims to elucidate possible relationships between dry bone weight and age:

Determine if bone weight (here a rudimentary proxy for bone density) is correlated with changes in age
Determine if relationships between dry bone weight and age vary between demographic groups (and particularly if females in this sample on average appear to have experienced greater changes in bone weight with age)
Determine if Bayesian linear modeling can produce accurate and precise estimates of age when the variables of innominate weight, biological sex, and innominate height are considered

## Materials and methods

### Sample and underlying patterns in the data

Innominates from 383 individuals from the Hamann-Todd Collection were examined for this study. The Hamann-Todd Collection is composed of over 3,000 skeletons, collected between the years 1912 and 1938, and is curated at the Cleveland Museum of Natural History in Cleveland, Ohio, USA [19]. Individuals aged 16–93 years were randomly selected from the collection, and even numbers of individuals (males and females from multiple population affinity groups) were selected from each decadal demographic group when possible to obtain a balanced sample (Table 1). Additionally, following the creation of the Bayesian linear model using this initial training sample (see heading below), this was tested on a small modern sample to assess model performance differences in a modern vs. historic sample. Data were collected from 30 (12 females and 18 males) individuals from the Forensic Anthropology Research Laboratory Donated Skeletal Collection at the Center for Forensic Science at Northern Michigan University in Marquette, Michigan, USA. The ages of these individuals at death ranged from 23–91 years (Table 2).

**Table 1:** Sample demographics for the training sample (category labels correspond to those assigned by the Hamann-Todd Collection).

Age	White Males	White Females	Black Males	Black Females	Total
16-20	7	6	14	12	39
21-30	17	18	11	16	62
31-40	10	11	12	14	47
41-50	11	12	11	14	48
51-60	9	10	10	17	46
61-70	10	10	21	15	56
71-80	19	20	15	7	61
81-93	6	9	4	5	24
Total	89	96	98	100	383

**Table 2:** Sample demographics for the test sample (category labels correspond to those assigned by the Northern Michigan University Donated Skeletal Collection).

Age	Asian	Unknown	European Males	European Females	American White Males	American White Females	Total
21-30	1	0	0	0	0	0	1
31-40	0	0	2	0	0	0	2
41-50	0	0	0	0	1	0	1
51-60	0	1	1	2	0	0	4
61-70	0	0	4	2	1	1	8
71-80	0	0	4	1	1	2	8
81-91	0	0	2	3	0	1	6
Total	1	1	13	8	3	4	30

Demographic information used in this study was assigned by the collections. Research permissions were obtained from the Hamann-Todd Collections Manager (Lyman Jellema) prior to data collection. The University of North Carolina at Chapel Hill Office of Human Research Ethics evaluated the Internal Review Board proposal submitted for this work and determined that it did not require IRB approval (Study #: 24-2470). All necessary permits were obtained for the described study, which complied with relevant regulations.

The left innominate was examined when it was undamaged; alternatively, the right innominate was examined when this criterion was not met. The weight of each innominate was recorded and rounded to the nearest whole gram.

**Fig 1:**
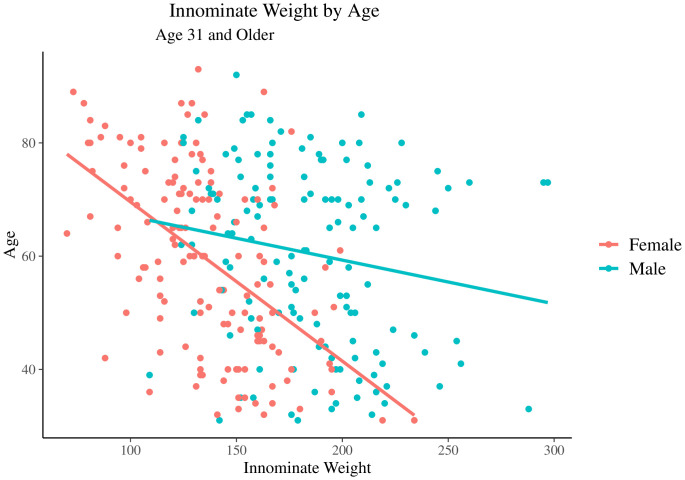
Dry bone weights (in grams) of innominates from individuals age 31+.

Dry bone weight patterns were examined visually to assess trends in the training sample, both throughout the lifespan and following the period of peak bone density in the late 20s. Fig 1 shows the variability in the data. Average dry bone weights were also examined via decadal age groups to determine the average peak of dry bone weight and when during the life span the decrease in weight appears. To assess correlation with age prior to the creation of predictive age estimation models, preliminary analyses were conducted. Pearson’s product-moment correlations for dry bone weight were completed for all individuals, all males, and all females. Average dry bone weights across each decadal age group were also computed. Average residuals from each decadal group were also calculated to assess trends in age estimation accuracy throughout the lifespan and are shown in Table 7. Statistical analyses were performed in R4.2.1 and in SAS 9.4 Statistical Software [20,21].

### Creation of a Bayesian linear model for estimating age from innominate dry bone weight

Following the observation of statistically significant correlations between innominate weight and age, as well as innominate height and weight, the authors created a Bayesian linear model for estimating age from the innominate. Because males and females exhibit different patterns of bone weight throughout the life span, sex is included in the model and is coded as a dummy variable. An interaction term with sex*innominate weight was included to account for these different slopes. Population affinity was explored as a predictor, but the accuracy and precision of the model was only negligibly different from the model without affinity. We therefore opted for the less complex model, and pooled population affinity into sex. Additionally, two models were created; one that encompasses individuals of all ages in the training sample and one for individuals 31 + . This second model was created to account for the growth patterns and corresponding peak in bone density in the late twenties, prior to the reduction in bone density following that time (see Results section).

In Bayesian linear regression, parameter estimates are drawn from the posterior probability distribution of the observed data, which is proportional to the product of the likelihood of the data and the prior probability of the parameters. In this study, the posterior predictive distribution was drawn with Markov Chain Monte Carlo sampling using Hamiltonian Monte Carlo and the NUTS sampling algorithm and a uniform prior using the rstanarm package in R [22]. A model was then fit to the simulated data, and these new parameter estimates were compared to the final model. The posterior distribution of the parameters and 95% credible intervals were computed from the final model. Model selection between competing models with different fixed effects was performed using leave one out cross validation (LOO-CV) [23] using the estimated log posterior density. Model precision was assessed using the Bayesian R^2^ [24] and accuracy was assessed using the median absolute value of the posterior residual distribution.

## Results

### Correlation between age and dry bone weight

In all demographic groups examined in the training sample (U.S. black and white males and females), a statistically significant correlation was found between known age and dry bone weight (α = 0.05). All correlations were negative, indicating that bone weight decreased with increasing age. R and p-values are displayed in Table 3. The *r* value represents the strength of the correlation, with values ranging from −1–1, denoting either a positive or negative association. Typically, |*r*| ≥ 0.7 indicates strong associations, 0.3 ≤ |*r*| < 0.7 indicates moderate associations, and |*r*| < 0.3 indicates weak associations [25].

**Table 3:** Pearson’s correlation coefficients between innominate dry bone weight by demographic group and age in the training sample.

Group	*r*	p-value
American White males	−0.356	<0.001
American Black males	−0.201	0.046
American White females	−0.596	<0.001
American Black females	−0.360	<0.001
All males in sample	−0.277	<0.001
All females in sample	−0.497	<0.001

While all groups in the training sample displayed a significant negative correlation between dry bone weight and age, these relationships were more pronounced among females than males. All males from the sample combined show a weak negative association with age, while all females combined exhibit a moderate negative association.

Average dry bone weights, displayed in Table 4, indicate that while both males and females in this sample exhibited a peak dry bone weight at approximately the same time (during the time that skeletal maturity is reached), females were subject to a more precipitous decrease in bone weight than males.

**Table 4:** Average innominate weight in grams by decadal age and demographic group in the training sample.

Age	White Males	White Females	Black Males	Black Females	All Males	All Females
16-20	221	161	204	156	212.5	158.5
21-30	199	165	201	155	200	160
31-40	182	161	212	160	197	160.5
41-50	183	158	212	148	197.5	153
51-60	170	132	180	145	175	138.5
61-70	175	126	183	130	179	128
71-80	184	116	199	128	191.5	122
81-93	153	108	179	124	166	116

Because clinical literature indicates that individuals may not reach peak bone mass until their late 20s, the 16–20 and 21–30 years of age individuals were removed from the analyses and all Pearson’s product-moment correlations were performed again (*n* = 282). Similarly to the results with all ages considered, all males combined in the 31 + age group in the training sample showed a weak negative correlation between innominate dry bone weight and age, and females showed a moderate negative correlation. Results for individuals 31 and older are summarized in Table 5.

**Table 5:** Pearson’s correlation coefficients between innominate dry bone weight by demographic group and age for individuals age 31 and older.

Group	*r*	p-value
American White males	–	0.205
American Black males	−0.236	0.044
American White females	−0.604	<0.001
American Black females	−0.361	0.001
All males	−0.202	0.018
All females	−0.506	<0.001

### Correlation between innominate weight and height

Because innominate weight throughout the life has been shown to be influenced by overall body size, the authors performed a Pearson’s product-moment correlation test to test for the association between innominate weight and height. Height of the innominate was measured to the nearest millimeter from the most superior point on the iliac crest to the most inferior point on the ischial tuberosity using an osteometric board (as outlined by Buikstra and Ubelaker [26]). With all individuals (e.g., both males and females) included, the results were statistically significant with a moderately strong relationship (p-value: < 0.001, *r* = 0.57). These results indicate that any predictive models for estimating age from innominate weight should also include innominate height measurements to control for variation in body size. Innominate dry bone weights and heights recorded for this study can be found in the Supplemental File associated with this manuscript (S1 File).

### Bayesian linear models

Out of the two models created, the Age 31+ model was more accurate than the All Ages mode (R^2^ = 0.33 and R^2^ = 0.32, respectively). While the R^2^ values were essentially the same, the accuracy and precision of the All Ages and Age 31+ models differed. This higher accuracy Age 31+ model had an average inaccuracy of 9.37 years. Estimated age was within ±10 years of known age for 57.3% of the training sample, and 87.2% of estimated ages at death were within ±20 years of known age. This is compared to the All Ages model, for which average inaccuracy was 11.96 years. Estimated age was within ±10 years of known age for 44.3% of the training sample, and 76.5% of estimated ages at death were within ±20 years of known age.

Model parameters for both the All Ages and Age 31+ models are shown in Table 6. Average residuals from each decadal group are shown in Table 7.

**Table 6:** Bayesian parameter estimates of the All Ages and Age 31+ models.

Variable	Parameter Estimate	SD	Lower 95% Credible Interval	Upper 95% Credible Interval
**All Ages Model**				
Intercept	−58.21	17.38	−91.99	−24.09
Sex (F = 0, M = 1)	−26.10	8.91	−43.52	−8.78
Innominate height	0.86	0.09	0.69	1.03
Innominate weight	−0.43	0.04	−0.51	−0.35
Sex * innominate height	0.16	0.05	0.06	0.27
**Age 31+ Model**				
Intercept	−10.82	15.89	−42.72	19.42
Sex (F = 0, M = 1)	−30.28	7.98	−45.82	−14.67
Innominate height	0.59	0.08	0.43	0.75
Innominate weight	−0.34	0.04	−0.41	−0.26
Sex * innominate height	0.20	0.05	0.11	0.29

**Table 7:** Average residuals for both the All Ages and Age 31 + models from each decadal group by sex.

Age	All Ages Model Males	Age 31+ Model Males	All Ages Model Females	Age 31+ Model Females
16-20	−20.95	–	−22.91	–
21-30	−21.86	–	−14.73	–
31-40	−9.99	−20.14	−3.97	−12.84
41-50	−2.54	−12.00	0.75	−8.02
51-60	2.04	−5.02	5.50	−1.25
61-70	10.69	4.34	10.88	5.14
71-80	21.24	13.93	10.02	6.75
81-93	23.35	19.26	21.53	17.54

When considering the performance of both models on the modern Northern Michigan University test sample, model inaccuracy for the Age 31+ model was 12.48, with 53% and 83% of the sample within 10 and within 20 years of known age, respectively. The age distribution for this sample was right-skewed, with a median age of 71, which was 10 years older than the Hamann-Todd training sample. Accordingly, the All Ages model did not perform as well, with an average inaccuracy of 14.81 years; 47% and 80% of predicted ages were within ±10 and ±20 years of known age, respectively.

## Discussion

The approach reported here is applicable only to skeletally mature individuals. While both the All Ages and Age 31+ models were generated and are reported here, the authors recommend only applying the Age 31+ model due to its higher performance in terms of both accuracy and precision in both the training and the test samples.

This age-at-death estimation method can be applied in practice by first weighing and measuring the innominate, obtaining an estimate or determination of skeletal sex (e.g., through DNA, metric, or morphological methods), and employing the appropriate equation (either the All Ages or Age 31 + model). The equations are as follows:

### Age 31+ model (recommended due to higher accuracy and precision)

Age in Years = −10.82 – (30.28 * Sex) + (0.59 * Innominate Height (in mm)) – (0.34 * Innominate Weight (in grams)) + (0.20 * Sex * Innominate Weight (in grams))

### All ages model (not recommended for use)

Age = -58.21 - (26.10 * Sex) + (0.86 * Innominate Height (in mm)) - (0.43 * Innominate Weight (in grams)) + (0.16 * Sex * Innominate Weight (in grams))

As an example of how to apply this method, consider one individual from the Hamann-Todd training sample (a 60 year old female with a dry bone weight of 128 grams and an innominate height of 198 mm). The Ages 31+ model equation would be used in the following way:

Age in Years = -10.82 - (30.28 * 0) + (0.59 * 198 mm) - (0.34 * 128 grams) + (0.20 * 0 * 128 grams)

This results in an age estimate of 62.48 years.

Predicted age in females was more accurate with these models than for males. The All Ages model performed poorly for individuals in younger age categories (under 30 years of age), which is not unexpected given that precipitous bone density loss typically does not occur at young ages. Perhaps unsurprisingly, this model also performed less well for individuals in older age categories, and particularly for males. The attenuated slopes of the model demonstrate that, similarly to many other aging methods, this method suffers from the perennial problem of “attraction to the middle” (i.e., overestimating the ages of younger individuals while underestimating the ages of older individuals).

As noted previously, in all analyses performed, females exhibit a decrease in dry bone weight with age which is more pronounced than that seen for males. For the Age 31 + model, where only individuals aged 31 years and older were included in the analyses, the strength of the correlation decreased in males but increased in females. These trends in dry bone weight, although imperfect proxies for bone mineral density, parallel trends observed in the clinical literature related to female bone density reduction with increasing age [11,12]. This decrease in bone mineral density puts particularly older females at an increased risk for bone fracture, especially after the age of 65, due to concomitant conditions such as osteoporosis and osteopenia [9].

With this study, the phenomenon of decreases in bone mineral density with age was observed and quantified through age-predictive Bayesian linear models. These results have broader implications for age-at-death estimation from skeletal remains. As noted previously, age-at-death estimation methods can be difficult to score consistently, particularly across observers of differing experience levels [27,28]. This method does not require access to advanced imaging techniques. This predictive model can be employed with standard osteology laboratory equipment (an osteometric board or sliding calipers, as detailed in *Standards*) and an inexpensive scale (such as a kitchen scale). Measurements of bone lengths have been shown to have low inter- and intra-observer error rates [29]. Additionally, this method can be performed far less subjectively than the other existing age estimation method that considers bone weight, TA3.

There are stipulations to using the regression equation detailed above. The first is that the innominate in question must be complete and relatively undamaged, as fragmentation affects weight. The second stipulation is that sex must be estimated from the innominate with fairly high confidence. The pubis is frequently cited as the most accurate area of the human body for sex estimation, and reliable resources exist for sexing the innominate [30–32]. It is notable that sex estimation methods from human bones estimate only biological sex (sometimes referred to as “osteological sex”) and do not represent social gender, nor are these methods capable of capturing all genetic and biological variation.

If biological sex can be estimated and the innominate is relatively undamaged, the bone may be weighed, measured, and evaluated for sex estimation, and this information can be input to the Bayesian linear model to produce a predicted age and 95% credible interval. The present study has shown that dry bone weights can be used to support estimations of chronological age; however, these analyses should be utilized in concert with other age-at-death estimation methods which have been validated with established accuracy rates.

While this exploratory study did see a correlation of dry bone weight with age, particularly in females as described in the clinical literature, it is important to consider the range of taphonomic influences which may have led to increased or decreased weight in dry bones, as these may affect interpretations and weights. For instance, maceration under certain conditions, such as boiling at high temperatures or with the use of corrosive chemicals, can cause bones to lose structural integrity and become lighter in weight than those processed without boiling or with the use of enzymes [33–36]. While records indicate that the remains in the Hamann-Todd Collection were macerated, specific maceration protocols that were utilized are unknown [37].

While collecting processing idiosyncrasies are important to consider when applying this method, the underlying structure of the reference data are also of import. For example, these results indicated a higher accuracy and precision on average in Hamann-Todd training data than in the test on individuals from the donated skeletal collection at the Center for Forensic Science at Northern Michigan University. This may be due to the restricted size in the latter collection; however, secular change may also be a factor, as well as the aforementioned differential processing and maceration procedures. Additionally, the test sample from Northern Michigan University skewed older on average than the reference data from the Hamann-Todd collection, which may bias method performance.

Consideration should also be given to the conditions under which the remains were recovered. The presence of residual organic content in bones persists in contexts with short postmortem intervals, but dry bone weight can decrease as a result of weathering and decomposition of organic content. Future research on dry bone weight correlation with age should be expanded to examine samples which are more modern and less historic than the Hamann-Todd Collection, such as donated body and forensic samples, which may present higher weights due to retention of oils and fats in the bone.

Furthermore, aging studies from bony indicators alone must be applied cautiously due to the numerous confounding variables which impact the skeleton throughout the lifespan. Mays [38] cites that up to 60% of age-related variation may be related to factors such as hormones, vitamin D status, and genetic and biomechanical factors which may not directly impact bony landmarks. While using dry bone weight as a proxy for bone mineral density does not address these factors, this study does utilize this measure in a step towards incorporating both clinical and osteological observations.

As this study has shown, decreased dry bone weight is associated with increased chronological age, especially among females. Although dry bone weight is only a proxy for bone mineral density, the straightforward, accessible, and inexpensive application of this method has promising implications for the use of dry bone weight in future age-at-death estimation studies.

## Supporting information

S1 File Supplemental data. Table with innominate height (in mm) and innominate weight (in grams) data collected for this study.
(XLSX)
